# Supplementary material for: Identification of photoperiod-induced specific miRNAs in the adrenal glands of Sunite sheep (Ovis aries)
Source: Front Vet Sci. 2022 Jul 22;9:888207. doi: 10.3389/fvets.2022.888207 (PMC9354845; doi:10.3389/fvets.2022.888207)
Supplement: Supplementary file 1 [file Data_Sheet_1.ZIP › Supplementary file/miRDeep2 file.zip!/miRDeep2 file/Know_Structure/SP42A2/oar-mir-3958.pdf]

miRBase precursor : oar-mir-3958
Total read count : 15972
oar-miR-3958-5p read count : 25
oar-miR-3958-3p read count : 15946
remaining reads : 1

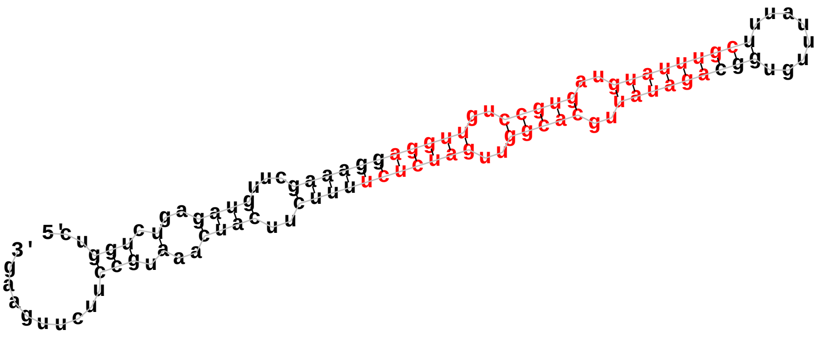

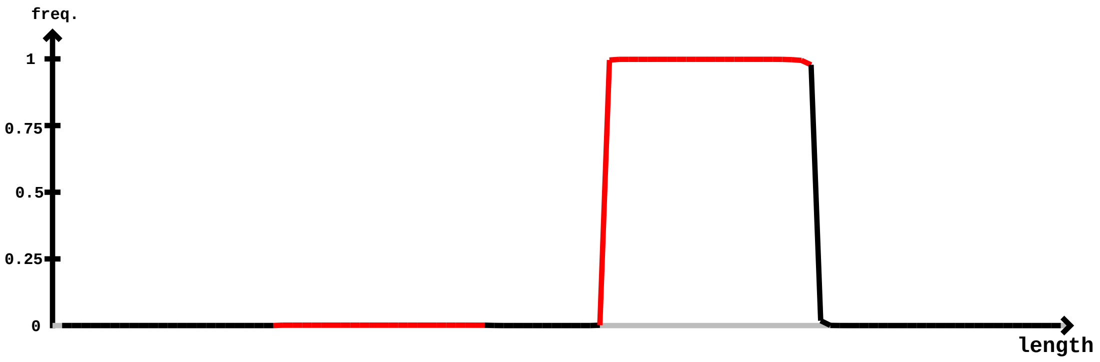


```
                                                        oar-miR-3958-3p
                      oar-miR-3958-5p
5'- cuggucugagauguucgaaaggagguuguccgugauguauuugcuuuauuuguggcagauauugcacgguugaucucuuuucuucaucaaaugccuucuugaag  -3'  exp
    ..(((.(..((((...((((((((((..(((((..(((((((((........)))))))))..)))))..))))))))))..))))..).))).........  reads   mm   sample
    cuggucugagauguucgaaagg..................................................................................  1       0    seq
    ......................agguuguccgugauguauuu..............................................................  1       0    seq
    ......................agguuguccgugauguauuug.............................................................  1       0    seq
    ......................agguuguccgugauguauuugc............................................................  17      0    seq
    ......................agguuguccgugauguauuugcu...........................................................  6       0    seq
    .......................................................cagauauugcacgguugaucu............................  2       0    seq
    .......................................................cagauauugcacgguugaucucu..........................  18      0    seq
    ........................................................agauauugcacgguugau..............................  4       0    seq
    ........................................................agauauugcacgguugauc.............................  18      0    seq
    ........................................................agauauugcacgguugaucu............................  40      0    seq
    ........................................................agauauugcacgguugaucuc...........................  264     0    seq
    ........................................................agauauugcacgguugaucucu..........................  15266   0    seq
    ........................................................agauauugcacgguugaucucuu.........................  283     0    seq
    ........................................................agauauugcacgguugaucucuuu........................  8       0    seq
    ........................................................agauauugcacgguugaucucuuuu.......................  1       0    seq
    .........................................................gauauugcacgguugaucu............................  1       0    seq
    .........................................................gauauugcacgguugaucuc...........................  1       0    seq
    .........................................................gauauugcacgguugaucucu..........................  37      0    seq
    .........................................................gauauugcacgguugaucucuu.........................  1       0    seq
    ..........................................................auauugcacgguugaucucu..........................  1       0    seq
    .............................................................uugcacgguugaucucu..........................  1       0    seq
```
